# A novel nomogram for predicting 3-year mortality in critically ill patients after coronary artery bypass grafting

**DOI:** 10.1186/s12893-021-01408-8

**Published:** 2021-11-30

**Authors:** HuanRui Zhang, Wen Tian, YuJiao Sun

**Affiliations:** Department of Geriatric Cardiology, The First Affiliated Hospital of China Medical University, NO.155 Nanjing North Street, Heping Ward, Shenyang, 110001 China

**Keywords:** Coronary artery bypass grafting, Mortality, Critically ill, Prediction model, Nomogram

## Abstract

**Background:**

The long-term outcomes for patients after coronary artery bypass grafting (CABG) have been received more and more concern. The existing prediction models are mostly focused on in-hospital operative mortality after CABG, but there is still little research on long-term mortality prediction model for patients after CABG.

**Objective:**

To develop and validate a novel nomogram for predicting 3-year mortality in critically ill patients after CABG.

**Methods:**

Data for developing novel predictive model were extracted from Medical Information Mart for Intensive cart III (MIMIC-III), of which 2929 critically ill patients who underwent CABG at the first admission were enrolled.

**Results:**

A novel prognostic nomogram for 3-year mortality was constructed with the seven independent prognostic factors, including age, congestive heart failure, white blood cell, creatinine, SpO_2_, anion gap, and continuous renal replacement treatment derived from the multivariable logistic regression. The nomogram indicated accurate discrimination in primary (AUC: 0.81) and validation cohort (AUC: 0.802), which were better than traditional severity scores. And good consistency between the predictive and observed outcome was showed by the calibration curve for 3-year mortality. The decision curve analysis also showed higher clinical net benefit than traditional severity scores.

**Conclusion:**

The novel nomogram had well performance to predict 3-year mortality in critically ill patients after CABG. The prediction model provided valuable information for treatment strategy and postdischarge management, which may be helpful in improving the long-term prognosis in critically ill patients after CABG.

**Supplementary Information:**

The online version contains supplementary material available at 10.1186/s12893-021-01408-8.

## Introduction

Coronary artery bypass grafting (CABG) is the most frequently performed operation in cardiac surgery [1], CABG has been the standard therapy for patients with left main or three-vessel coronary artery disease [2]. With the development of surgery technology and improvement of nursing quality, the operative mortality, operative complication, and in-hospital mortality have decreased significantly [1, 3, 4]. As many adverse clinical events occur after discharge, the long-term outcomes for patients after CABG have been received more and more concern [4, 5]. Accurate assessment of long-term mortality risk in patients after CABG is very important for clinicians to make individualized treatment and management strategy, this will bring more benefit for patients after CABG, thereby reducing its mortality.

In order to assess cardiac operative risk, a variety of risk score systems are widely used, in which European system for cardiac operative risk evaluation (EuroSCORE) and Society of Thoracic Surgeons (STS) are the most widely used [6, 7]. But long-term mortality prediction model for patients after CABG is still lack in clinic. It is really important to find a risk prediction model of long-term mortality, thereby improving long-term outcomes in patients after CABG. Nomogram is a more simple and convenient method for predicting clinical outcomes by giving a score to potential risk factors [8]. Recently, nomogram has been increasingly applied in evaluating prognosis of various diseases, such as tumor [9], myocardial infarction [10], acute renal failure [11], acute pancreatitis [12] and so on.

In the study, based on a publicly Medical Information Mart for Intensive cart III (MIMIC-III), we firstly identify the risk factors of 3-year mortality in critically ill patients after CABG, and then further develop a prognostic nomogram for predicting 3-year mortality in these patients. Finally, the accuracy of prognosis nomogram is verified by validation cohort.

## Methods

### Database

We developed the prediction model by extracting data from MIMIC-III, v1.4 [13], which is an openly available database contains information of 46520 critically ill patients who received treatment in intensive care unit (ICU) of Beth Israel Deaconess Medical Center from 2001 to 2012. After successful application (certification number: 37650993) and approved by the institutional review boards (IRB) of the Massachusetts Institute of Technology (MIT) and Beth Israel Deaconess Medical Center (BIDMC), we were granted access to the database and utilized the data. Because unidentified health data of patients was used, informed consent was waived by both IRB of MIT and BIDMC. And all procedures in our study were in accordance with the corresponding guidelines.

### Participant selection and data extraction

We included critically ill patients who underwent CABG at this admission according to ICD-9 code. We excluded the patients as follows: (1) multiple admission; (2) inappropriate age (< 18 or > 89 years old); (3) length of stay in ICU < 24 h; (4) follow-up time < 3 years. The primary endpoint is 3-year mortality in this study.

All relevant clinical data was extracted within the first 24 h after ICU admission using Structured Query Language (SQL). For the model development, we retrospectively collected the following data: (1) Demographic data: age and gender; (2) Comorbidities: diabetes, previous myocardial infarction, previous stroke, congestive heart failure (CHF) and renal failure; (3) 24 h vital signs: systolic blood pressure (SBP), diastolic blood pressure (DBP), heart rate (HR), respiratory rate and mean blood pressure (MBP); (4) Laboratory parameters: hemoglobin, white blood cell (WBC), lactate, anion gap, phosphoric acid, activated partial thromboplastin time (APTT), blood urea nitrogen (BUN), alanine aminotransferase (ALT), glutamic oxalacetic transaminase (AST), phosphoric acid (PA), creatinine, platelet, potassium, and sodium, PCO_2_, PO_2_, SpO_2_, and pH; (5) Management in hospital: mechanical ventilation, continuous renal replacement treatment (CRRT), and vasopressor use; (6) Scoring systems: the Oxford Acute Severity of Illness Score (OASIS), the Sequential Organ Failure Assessment (SOFA), and the Simplified Acute Physiology Score II (SAPS II), which were calculated within the first 24 h after ICU admission.

### Missing data management

For the model development, we excluded lactate, AST, ALT and PA because of the portion of the missing value > 20% (Additional file 1). For other variables with missing value < 20%, missing values of variables were filled by a multiple imputation method, which could reduce the bias caused by missing values [14].

### Statistical analysis

Continuous data were reported as median and inter quartile range and compared by Kruskal–Wallis H test. Categorical data were presented as count and percentage and compared using Pearson’s χ^2^ test or Fisher’s exact test as appropriate. The objective of this study was to develop an easy-use prediction model for 3-year mortality in critically ill patients. For the model development, the univariate and multivariable logistic regression were implemented to screen independent predictors in the primary cohort. The variables with P < 0.05 in univariate logistic regression were selected into the following analysis. For identifying the final prediction model, a backward step-selection method with the Akaike information criterion (AIC) was used to select predictors in multivariable logistic regression. The “mice” package was used to multiple imputation for variables with less than 20% missing values [14, 15]. The “rms” package was applied for plotting nomogram and calibration curve. The Receiver Operating Characteristic (ROC) curve was performed to assess discrimination ability of the nomogram for the 3-year mortality using “pROC” package [16]. DeLong’s non-parametric approach was implemented to compare differences of the area under the curve (AUC) between model and other traditional scoring systems [17]. Calibration slope and the Brier value were performed to evaluate the calibration of the model [18]. Bootstrapping with 1000 resamples was used for calibration analysis. The decision curve analysis (DCA) was used to evaluate the clinical practicability of the nomogram by quantifying the standardized net benefits at various threshold probabilities using the “rmda” package [19]. A two-sided P value of < 0.05 was considered statistically significant. We performed all statistical analyzes using R software (version 4.0.3).

## Results

### Baseline characteristics of the primary and validation cohort

**Fig. 1:**
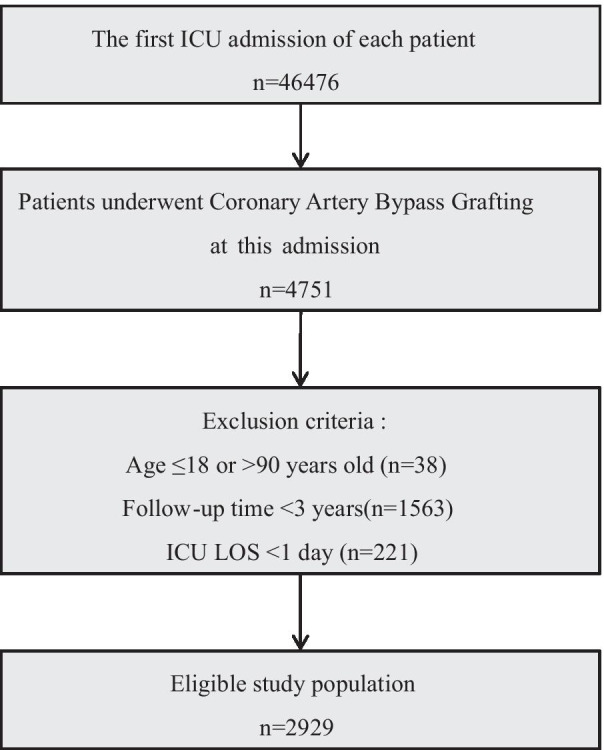
The flow chart of participant selection

A total of 2929 patients who underwent CABG were included into the final cohort after the screening by the inclusion and exclusion criteria (Fig. 1). We assigned 2050 patients (265 deaths, 3-year mortality rate 12.9%) to the primary cohort and 879 patients (113 deaths, 3-year mortality rate 12.9%) to the validation cohort. All baseline characteristics of the primary and validation cohort are shown in Table 1. There were no obviously statistical differences between the primary and validation cohort. For the following model development, we excluded lactate, AST, ALT, and PA because of the portion of the missing value > 20% (Additional file 1).

**Table 1:** Characteristics of the study patients between the primary cohort and the validation cohort

Variables	Total	Primary cohort	Validation cohort	P value
	(n = 2929)	(n = 2050)	(n = 879)	
Basic demographics				
Age, years	68.6 (60.1–76.2)	68.6 (60.0–76.2)	68.9 (60.3–76.4)	0.375
Gender, female	781 (26.7)	539 (26.3)	242 (27.5)	0.516
Comorbidities, n (%)				
CHF	764 (26.1)	540 (26.3)	224 (25.5)	0.661
Renal failure	209 (7.1)	153 (7.5)	56 (6.4)	0.330
Diabetes	1110 (37.9)	791 (38.6)	319 (36.3)	0.258
Previous myocardial infarction	551 (18.8)	385 (18.8)	166 (18.9)	0.988
Previous stroke	40 (1.4)	34 (1.7)	6 (0.7)	0.056
24 h vital signs				
Mean SBP, mmHg	111.4 (105.8–119.0)	111.4 (105.8–119.1)	111.5 (106.0–118.8)	0.542
Mean DBP, mmHg	56.1 (52.6–60.5)	56.1 (52.6–60.4)	56.3 (52.6–60.6)	0.626
Mean HR, beats/min	85.2 (79.4–91.1)	85.3 (79.5–91.1)	85.0 (78.9–91.2)	0.266
Mean respiratory rate, beats/minute	16.7 (15.0–18.8)	16.7 (15.0–18.7)	16.7 (15.1–18.9)	0.528
Mean MBP, mmHg	74.3 (70.8–79.0)	74.3 (70.6–78.9)	74.4 (71.0–79.2)	0.425
Laboratory parameters				
Hemoglobin, g/dL	10.0 (8.9–11.2)	10.1 (8.9–11.3)	10.0 (8.9–11.2)	0.473
WBC, 10^9^/L	11.9 (9.2–15.4)	12.0 (9.2–15.5)	11.6 (9.0–15.2)	0.300
Lactate, mg/dL	2.2 (1.5–3.2)	2.3 (1.6–3.2)	2.1 (1.5–3.1)	0.043
Anion gap, mmol/L	12.0 (11.0–14.0)	12.0 (11.0–14.0)	12.0 (11.0–14.0)	0.650
Phosphoric acid, mg/dL	3.5 (2.9–4.1)	3.4 (2.8–4.0)	3.5 (3.0–4.2)	0.025
APTT, second	35.5 (30.6–44.7)	35.5 (30.5–44.7)	35.4 (30.7–44.6)	0.631
BUN, mg/dL	16.0 (12.0–20.0)	15.0 (12.0–20.0)	16.0 (12.0–20.0)	0.884
ALT, U/L	25.5 (17.0–39.8)	24.0 (17.0–40.5)	26.0 (17.0–38.0)	0.767
AST, U/L	44.0 (26.0–92.5)	44.0 (26.0–91.0)	46.0 (27.0–99.0)	0.516
PA, mg/dL	3.5 (2.9–4.1)	3.5 (3.0–4.2)	3.4 (2.8–4.0)	0.025
Creatinine, U/L	0.8 (0.7–1.0)	0.8 (0.7–1.0)	0.8 (0.7–1.0)	0.742
Platelet, 10^9^/L	158.0 (122.0–204.0)	160.0 (123.0–205.0)	156.0 (120.5–199.5)	0.166
Potassium, mmol/L	4.3 (3.9–5.0)	4.3 (3.9–5.0)	4.3 (3.9–5.0)	0.733
Sodium, mmol/L	137.0 (135.0–139.0)	137.0 (135.0–139.0)	137.0 (135.0–138.0)	0.081
PCO_2_ (mmHg)	40.0 (37.0–45.0)	40.0 (37.0–45.0)	40.0 (37.0–44.0)	0.816
PO_2_ (mmHg)	338.0 (261.0–403.0)	335.0 (259.0–400.0)	345.0 (268.2–410.8)	0.060
SpO_2_ (%)	97.0 (78.0–98.0)	97.0 (79.0–98.0)	97.0 (78.0–98.0)	0.169
pH	7.4 (7.4–7.4)	7.4 (7.4–7.4)	7.4 (7.4–7.4)	0.726
In-hospital management, n (%)				
Mechanical ventilation	2771 (94.6)	1940 (94.6)	831 (94.5)	0.988
RRT	46 (1.6)	34 (1.7)	12 (1.4)	0.672
Vasopressor use	2473 (84.4)	1733 (84.5)	740 (84.2)	0.854
1-year mortality, n (%)	203 (6.9)	137 (6.7)	66 (7.5)	0.467
3-year mortality, n (%)	378 (12.9)	265 (12.9)	113 (12.9)	1.000

*CHF* congestive heart failure; *SBP* systolic blood pressure; *DBP* diastolic blood pressure; *HR* heart rate; *MBP* mean blood pressure; *WBC* white blood cell; *APTT* activated partial thromboplastin time; *BUN* blood urea nitrogen; *ALT* alanine aminotransferase; *AST* glutamic oxalacetic transaminase; *PA* phosphoric acid; *RRT* renal replacement treatment; *OASIS* Oxford Acute Severity of Illness Score; *SOFA* Sequential Organ Failure Assessment; *SAPS II* Simplified Acute Physiology Score II

### Model development in the primary cohort

**Fig. 2:**
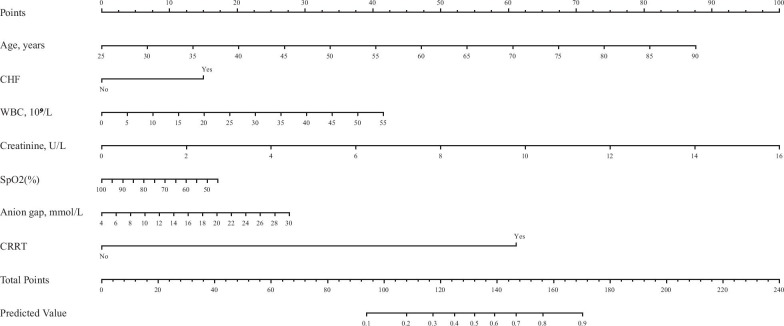
Nomogram to calculate risk score and predict 3-year mortality in patients underwent CABG at this admission. The nomogram was developed in the primary cohort, with age, CHF, WBC, creatinine, SpO_2_, anion gap, and CRRT

Baseline demographics, comorbidities, vital signs, laboratory parameters, and in-hospital management for the prediction of 3-year mortality were examined by the univariate logistic regression (Additional file 2). The age, gender, CHF, renal failure, mean DBP, hemoglobin, WBC, APTT, creatinine, BUN, SpO_2_, anion gap, sodium, and CRRT were potential prognostic factors of 3-year mortality (P < 0.05) (Table 2). All the above predictors were entered into the multivariable logistic regression. The age, CHF, WBC, creatinine, SpO2, anion gap, and CRRT were selected as the independent predictors for 3-year mortality in the final prediction model (P < 0.05 of each predictor) (Table 2). The VIF was calculated and there was no significant multicollinearity in the model (VIF < 2). Furthermore, the correlation between continuous variables and outcome was visualized by loess curves in Additional file 3. A prognostic nomogram for 3-year mortality was plotted with the seven prognostic factors derived from the the multivariable logistic regression (Fig. 2).

**Table 2:** Univariate and multivariable analyses for the relationship between the candidate risk factors and 3-year mortality in the primary cohort

Variables	Univariate		Multivariate	
	OR (95% CI)	P	OR (95% CI)	P
Age, years	1.07 (1.05–1.08)	< 0.001	1.08 (1.06–1.10)	< 0.001
Gender, female	1.38 (1.05–1.83)	0.022		
CHF	3.74 (2.87–4.87)	< 0.001	2.37 (1.76–3.18)	< 0.001
Renal failure	3.29 (2.27–4.78)	< 0.001		
Mean DBP, mmHg	0.96 (0.94–0.98)	0.001		
Hemoglobin, g/dL	0.92 (0.85–0.99)	0.023		
WBC, 10^9^/L	1.03 (1.00–1.05)	0.018	1.04 (1.02–1.07)	0.001
APTT, second	1.02 (1.01–1.02)	< 0.001		
Creatinine, U/L	1.55 (1.36–1.77)	< 0.001	1.43 (1.24–1.66)	< 0.001
BUN, mg/dL	1.05 (1.04–1.06)	< 0.001		
SpO2 (%)	0.97 (0.96–0.98)	< 0.001	0.98 (0.97–0.99)	< 0.001
Anion gap, mmol/L	1.18 (1.13–1.24)	< 0.001	1.06 (1.01–1.12)	0.024
Sodium, mmol/L	0.96 (0.92–1.00)	0.048		
CRRT	43.75 (16.77–114.11)	< 0.001	33.53 (12.63–107.10)	< 0.001

Each prognostic factor was assigned various weighted score in the nomogram. The values of age, WBC, creatinine, SpO_2_, and anion gap ranged from 25 to 90, 0 to 55, 0 to 16, 45 to 100, and 4 to 30, respectively. The highest total score was 240 points, and the scale of the 3-year mortality probability ranged from 0.1 to 0.9. If a patient who underwent CABG had an age of 68 years old, HF, a WBC value of 7.3*10^9^/L, a creatinine value of 7.6 U/L, a SpO_2_ value of 78%, an AG value of 20 mmol/L, and without underwent CRRT, the 3-year mortality probability was 74.3%.

### Model performance

**Fig. 3:**
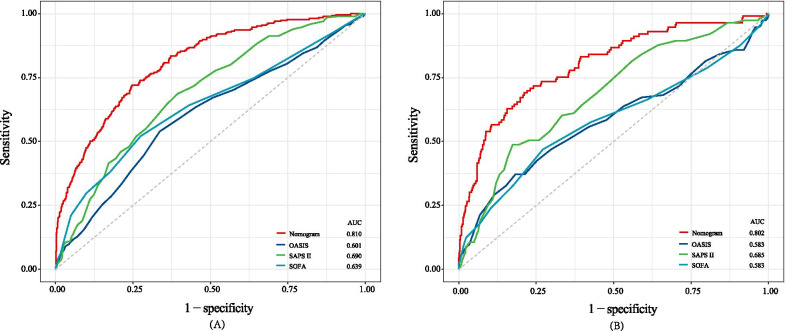
Receiver operating characteristic curves of the nomogram and traditional scoring systems in primary (**A**) and validation (**B**) cohorts

**Fig. 4:**
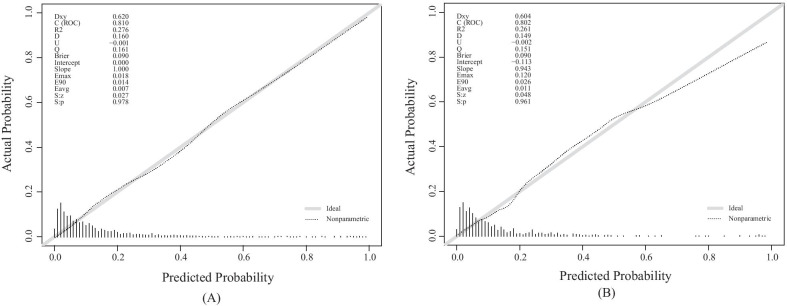
Calibration curve of nomogram in the primary (**A**) and validation (**B**) cohorts. The horizontal axis represents the predicted probability of 3-year mortality, and the vertical axis represents the actual observed 3-year mortality

**Fig. 5:**
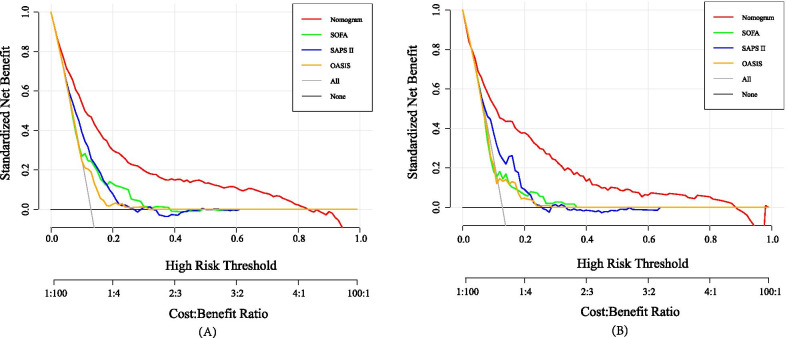
Decision curves for the primary (**A**) and validation (**B**) cohorts, implicating the net benefit with respect to the use of the nomogram and traditional scoring systems. X-axis and y-axis represent threshold probability and net benefit, respectively

The ROC curves indicated that the nomogram (AUC: 0.810) had the good predictive capacity, which was greater than SAPSII (AUC: 0.690), SOFA (AUC: 0.639), and OASIS (AUC: 0.601) in the primary cohort (All P value < 0.001) (Fig. 3A). Meanwhile, the similar predictive performance (AUC: 0.802) was found in the validation cohort, which were greater than traditional scoring systems (SAPSII, AUC: 0.685; SOFA, AUC: 0.583; OASIS, AUC: 0.583; All P value < 0.001) (Fig. 3B). The calibration plot indicated the nomogram had adequate fit for 3-year mortality in primary (Brier score: 0.090, calibration slope: 1.000) and validation cohort (Brier score: 0.090, calibration slope: 0.943), respectively (Fig. 4A, B). The decision curve analysis (DCA) showed that this nomogram had a large threshold probability range than the SAPSII, SOFA, and OASIS. And, at the same threshold probability, this nomogram showed higher net benefit than SAPSII-, SOFA-, and OASIS-assisted decisions in primary and validation cohort, respectively (Fig. 5A, B).

## Discussion

The present study developed and internally validated a novel prognosis nomogram model based on MIMIC-III database to predict 3-year mortality in critically ill patients after CABG. Seven risk factors for 3-year mortality of these patients were identified by logistic regression method, including age, CHF, WBC, Creatinine, SpO2, anion gap and CRRT. The prediction ability of the novel prognosis nomogram was evaluated by AUC, calibration curve analysis and decision curve analysis in development and validation cohort, and the results found the novel prognosis nomogram model had fine stability and precise prediction ability, which was significantly superior to SOFA, OASIS and SAPSII.

A large difference is observed in severity, progress and prognosis of disease on patients after CABG. Many predictive score systems for the risk of cardiac operative have been created over decades, such as EuroSCORE [6], STS [7] and EuroSCORE II [20]. These predictive score systems not only need more information, but also is complicated and difficult to acquired rapidly. Meanwhile, these score systems are not designed for predicting long-term mortality risk in patients after CABG. The population of MIMIC III database was from critically ill patients in ICU. It is widely known that the severity score systems including SOFA, OASIS, SAPSII are typically used for risk stratification of critically ill patients, and have good prediction ability for predicting the outcome of patients with critically ill [21–23]. In the study, the prediction ability of novel nomogram was better than the severity score systems (SOFA, OASIS, SAPSII) for predicting 3-years mortality in critically ill patients after CABG. The novel nomogram model contained only seven accessible factors but had better prediction ability and calibration in the present study, so the novel nomogram may be worth generalizing extensively in clinical application.

Recent studies on prediction model for prognosis of patients after CABG has been developed. The research developed that a model of predicting hospital readmission in patients after CABG, 30-days all cause readmission can be predicted by the model [24]. Some studies developed predictive model of renal disease among patients after CABG. A nomogram model based on 7 predictors provided reliable prediction ability of acute kidney injury in heart failure patients after CABG [25]. The ACHE score was end-stage renal disease prediction model follow CABG with a long-term follow-up, which had advantages in simplicity and preciseness [26]. In order to improve the nursing quality and make individualized clinical decision, several models of predicting ICU length of stay after CABG have been developed [27, 28]. A study built up machine learning models to predict 30-day mortality and three complications in critically ill patients after open-heart surgery (including CABG) from MIMIC III database [29]. The machine learning model predicted the short-term outcome by window 10 software with more than 30 risk factors. However, our nomogram model directly predicted 3-year mortality in critically ill patients after CABG by seven risk factors. The machine learning model and our nomogram model were from MIMIC III database, but their predictors and predicting outcome were different. Previous study established long-term survival prediction model after CABG in 31–90 days, 91–365 days, 1–3 years and > 3 years, respectively, these four times intervals model shared thirteen common risk factors [30]. However, our nomogram model based on critically ill patients to predict 3-year mortality after CABG, which was different from that long-term survival prediction model. The long-term survival prediction model at 4 distinct time intervals suggested the effect of thirteen risk factors on mortality after CABG may be different at different points in time. But the long-term mortality risk should be rapidly assessed in order to early risk stratification in clinical practice, which may provide clinician important clues for individualized treatment strategies to improve prognosis in critically ill patients after CABG. In fact, our novel nomogram model has the advantages of convenience, exactness and high efficiency, can be a satisfactory model for predicting 3-year mortality in critically ill patients after CABG.

The predictors of novel nomogram model are common and easily accessible clinical parameters, and associated with prognosis after CABG. Age and CHF were acknowledged as the risk factors influencing the prognosis of patients after CABG [6, 7, 20, 31]. WBC play an essential role in cardiovascular disease, some studies have found elevated WBC was associated with cardiovascular complications and mortality in patients after CABG [29, 32]. Creatinine increased during perioperative stage served as independent risk factor for mortality in patients after CABG, and some risk assessment models after cardiac surgery included creatinine [6, 20, 33, 34]. SpO_2_ as an earlier warning index of hypoxemia is very important prognostic factor for critically ill patients with cardiovascular disease [35, 36]. Recent studies have found anion gap is correlated with cardiovascular disease and a useful indicator in assessing risk stratification in critically ill patients [37–39]. CRRT was commonly used in critically ill patients in ICU, critically ill patients requiring CRRT had a greater risk of hospital and post-discharge mortality in ICU [40, 41]. Above all, the seven risk factors of our novel nomogram are generally available and widely supported in clinical application.

There still exist some limitations in the present study. The study was based on MIMIC III database of critically ill patients in a single center, and internal verification was carried out to validate the performance of nomogram model, it may be not suitable to generalize the nomogram model based on critically ill patients after CABG to all patients after CABG, and could not be considered as a preoperative assessment of the patients. But the nomogram model will be more helpful for the ICU team to assess the prognosis for critically ill patients after CABG. With the development of medical technology and the improvement of the quality of nursing, the mortality of patients after CABG has decreased in recent years, but all patients underwent CABG from 2001 to 2012 in the study, some new potential confounding factors may affect the performance of prognosis nomogram. In addition, some patients after CABG were not enrolled due to incomplete data and some importance data was missing that might affect the mortality of patients after CABG in the study, which may cause bias of the results.

In conclusion, the novel developed nomogram showed well performance as a prediction model of 3-years mortality in critically ill patients after CABG, and consisted of simple seven clinical variables, which might be widely applied in risk stratification of long-term mortality for these patients in ICU. The novel nomogram gave valuable information to help clinician for decision making in treatment and management of critically ill patients after CABG, and these patients would benefit from strengthen postdischarge management and close follow-up.

## Supplementary Information

**Additional file 1.** The histogram and pattern of missing data.

**Additional file 2.** Univariate analyses for the relationship between the candidate risk factors and 3-year mortality in the primary cohort.

**Additional file 3.** Loess curves for the correlation between continuous variables and 3-year mortality.

## Data Availability

All data presented in the study are available in the MIMIC III database.
