# Supplementary figures and images for: A novel nomogram for predicting 3-year mortality in critically ill patients after coronary artery bypass grafting
Source: BMC Surg. 2021 Nov 30;21:407. doi: 10.1186/s12893-021-01408-8 (PMC8638264; doi:10.1186/s12893-021-01408-8)

# Histogram of missing data

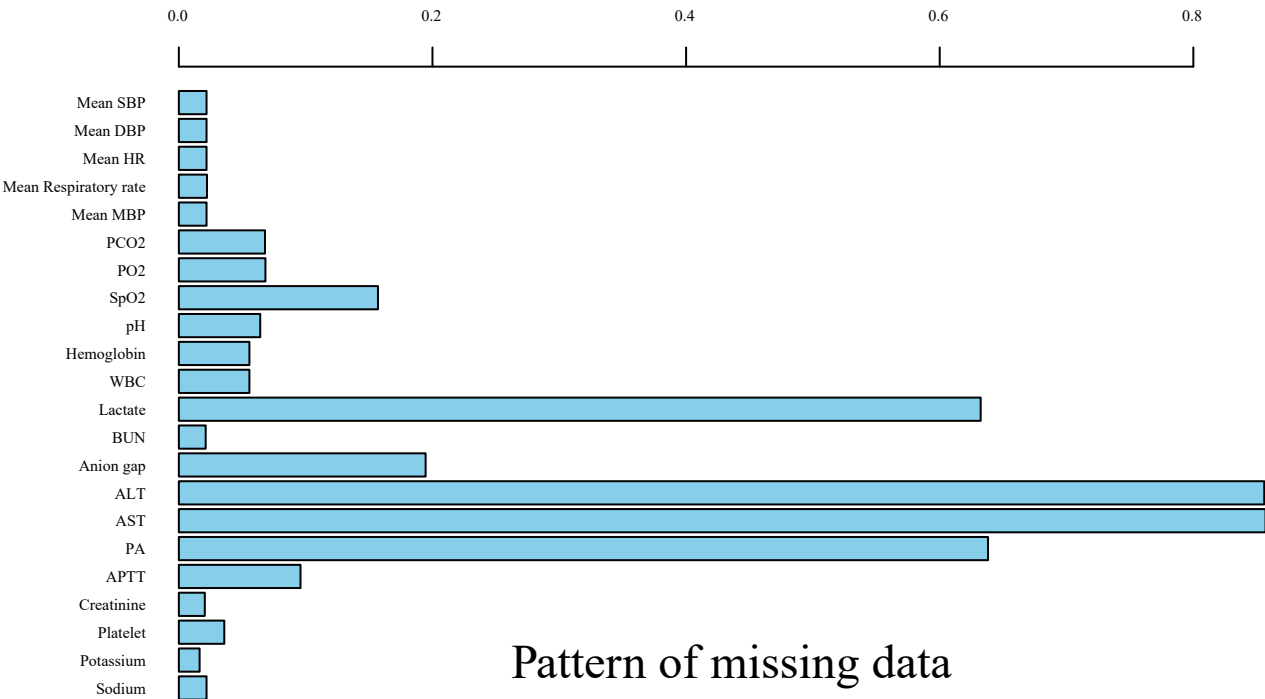

# Pattern of missing data

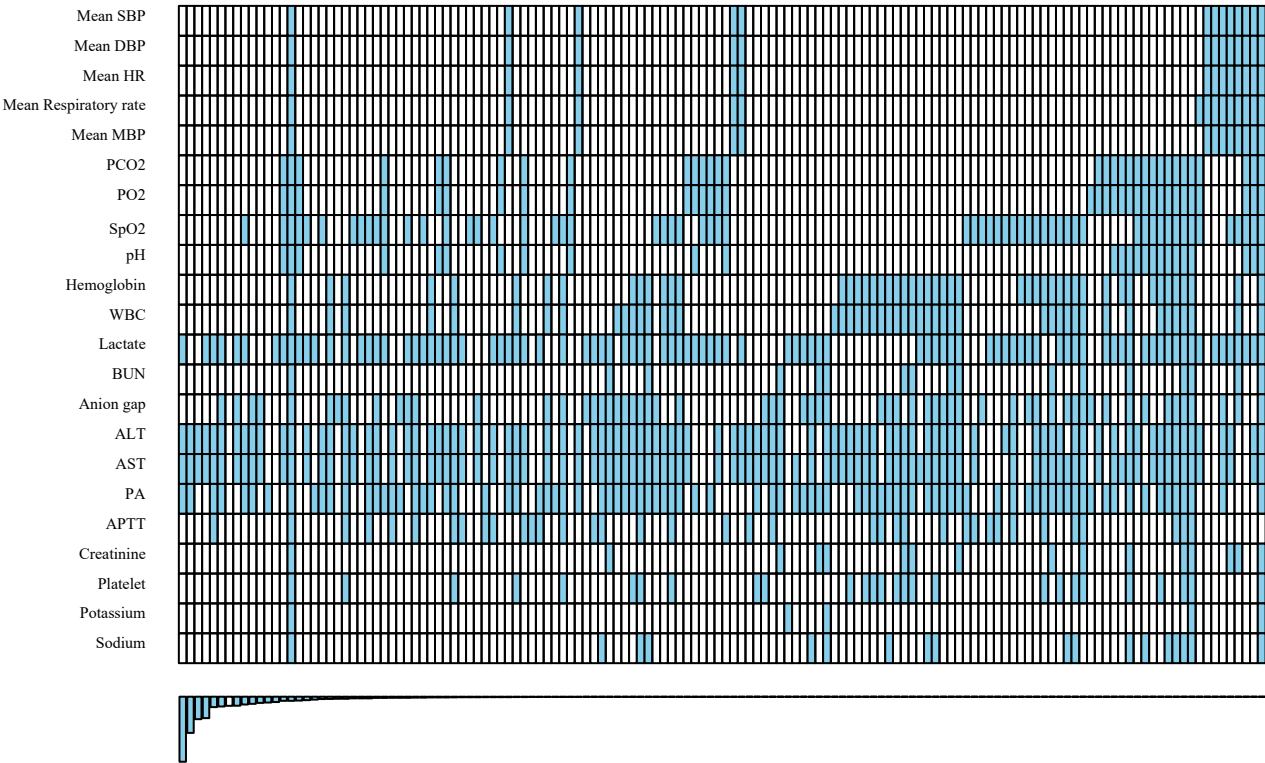

Supplement: Supplementary file 1 — Additional file 1. The histogram and pattern of missing data. [file 12893_2021_1408_MOESM1_ESM.pdf]

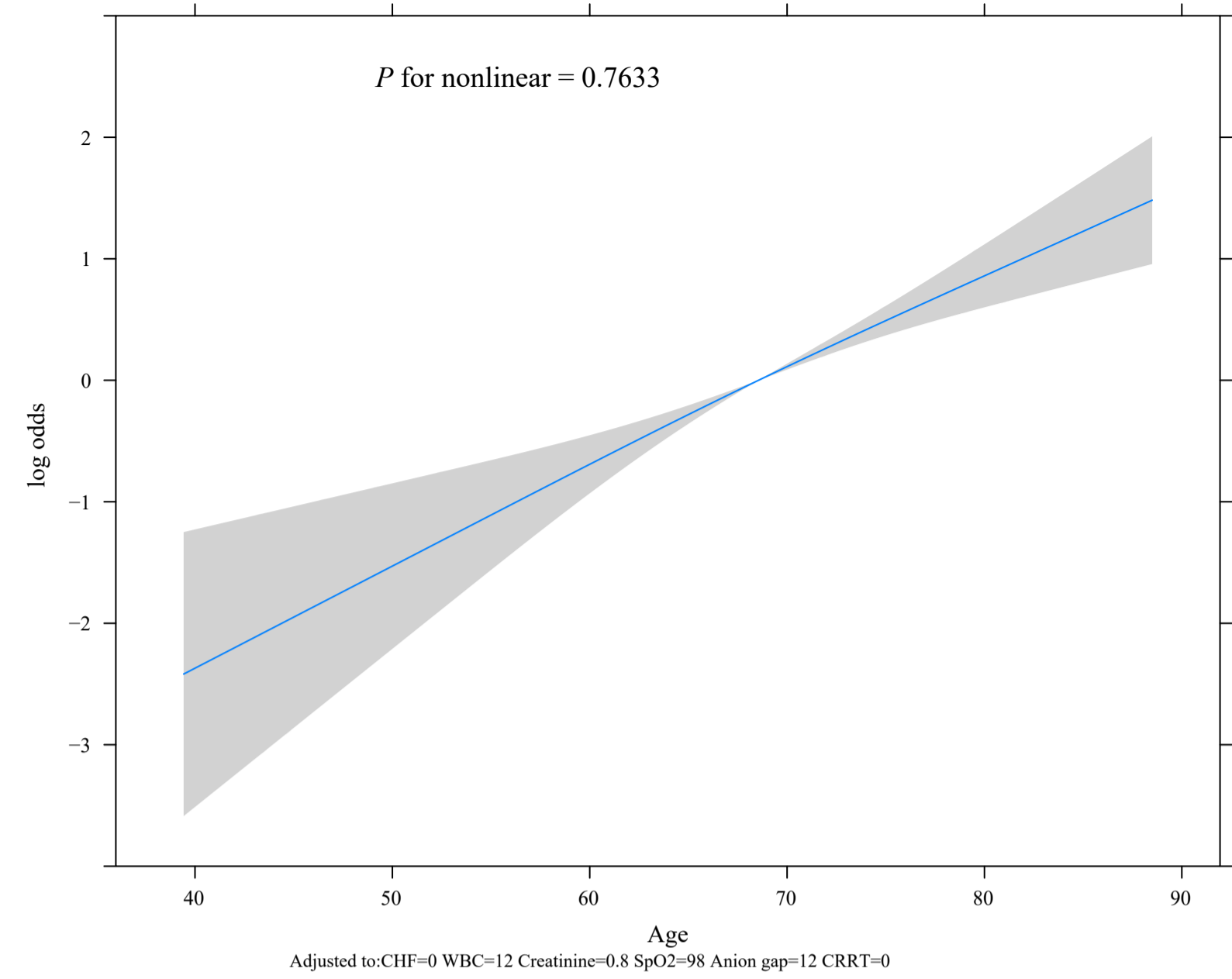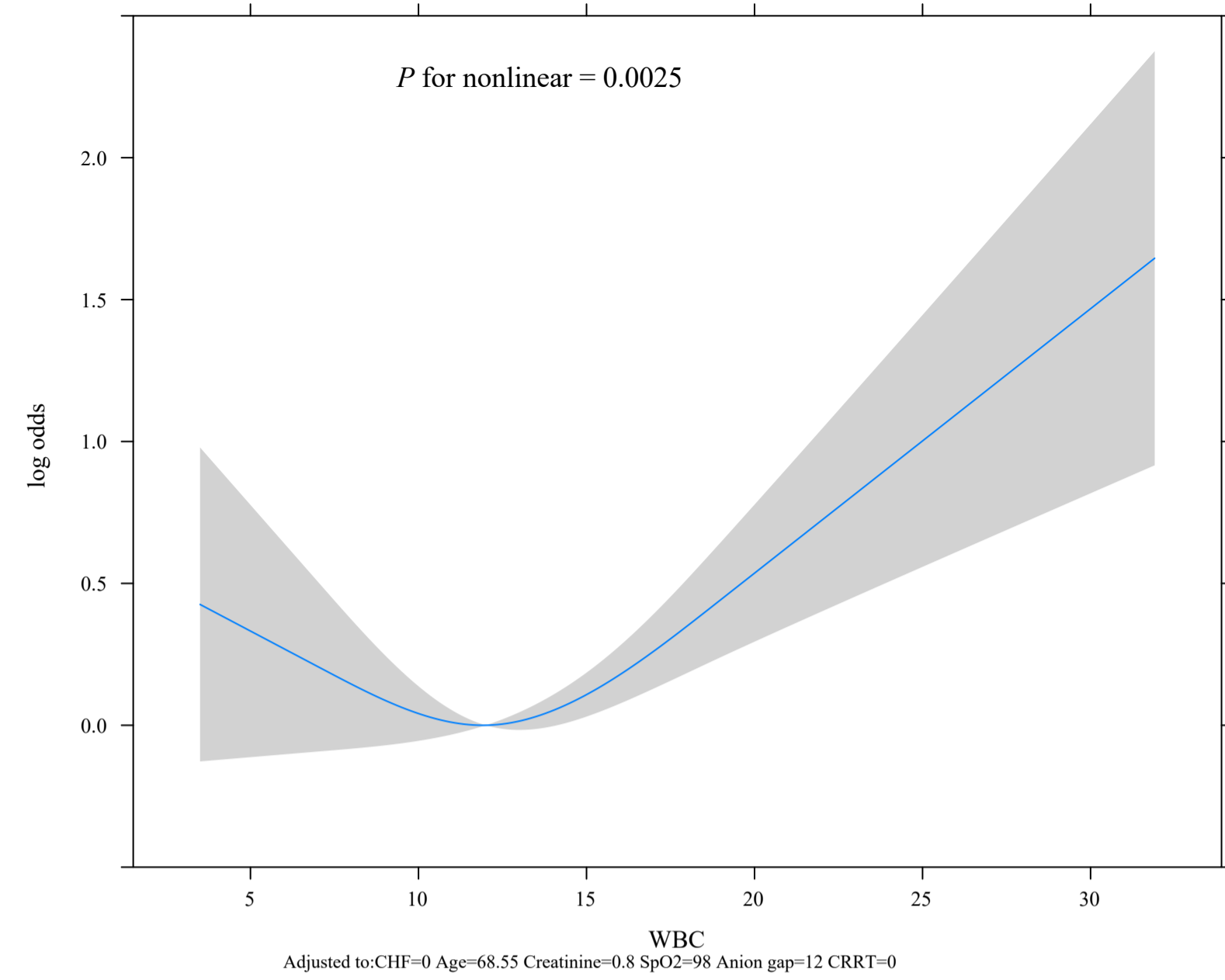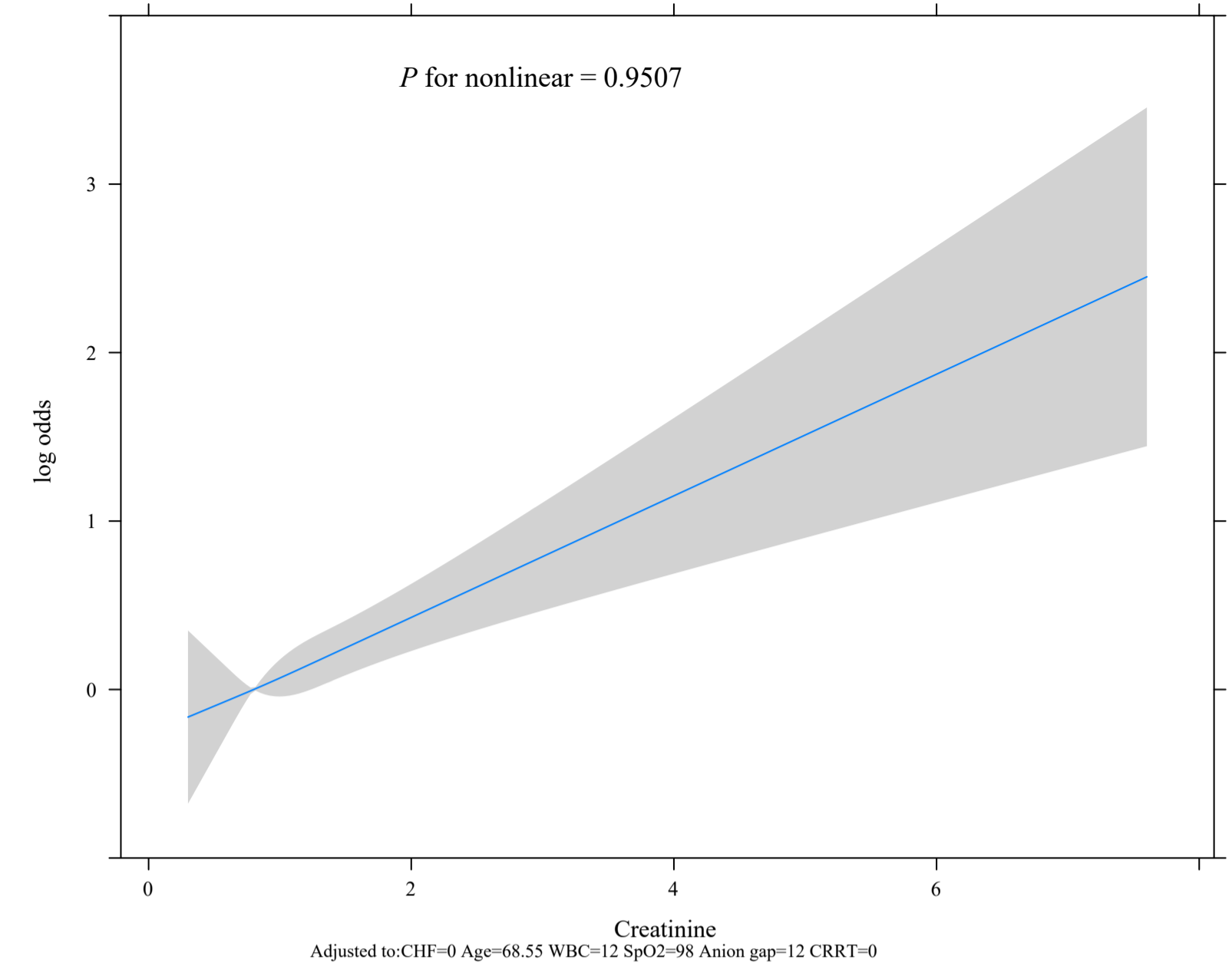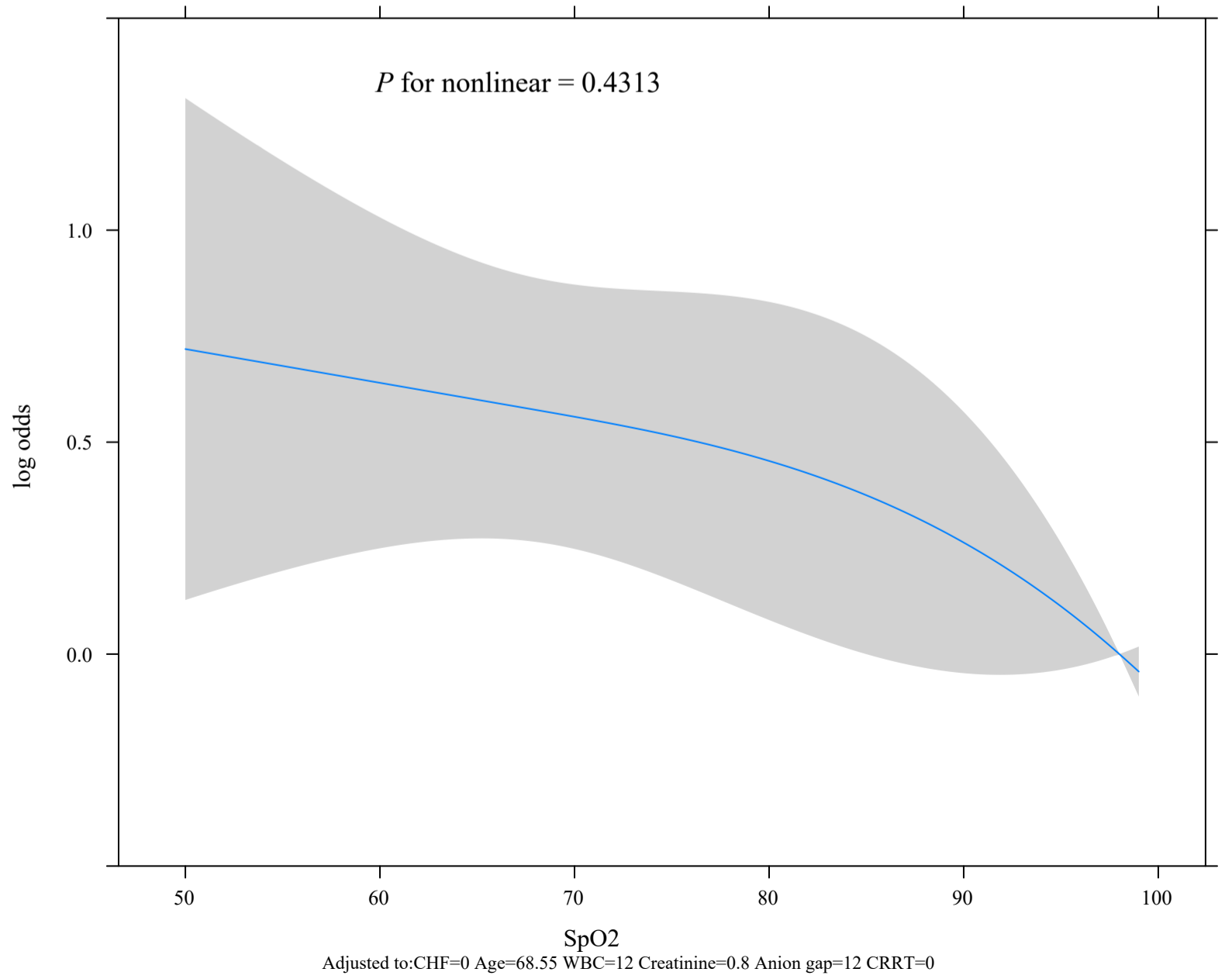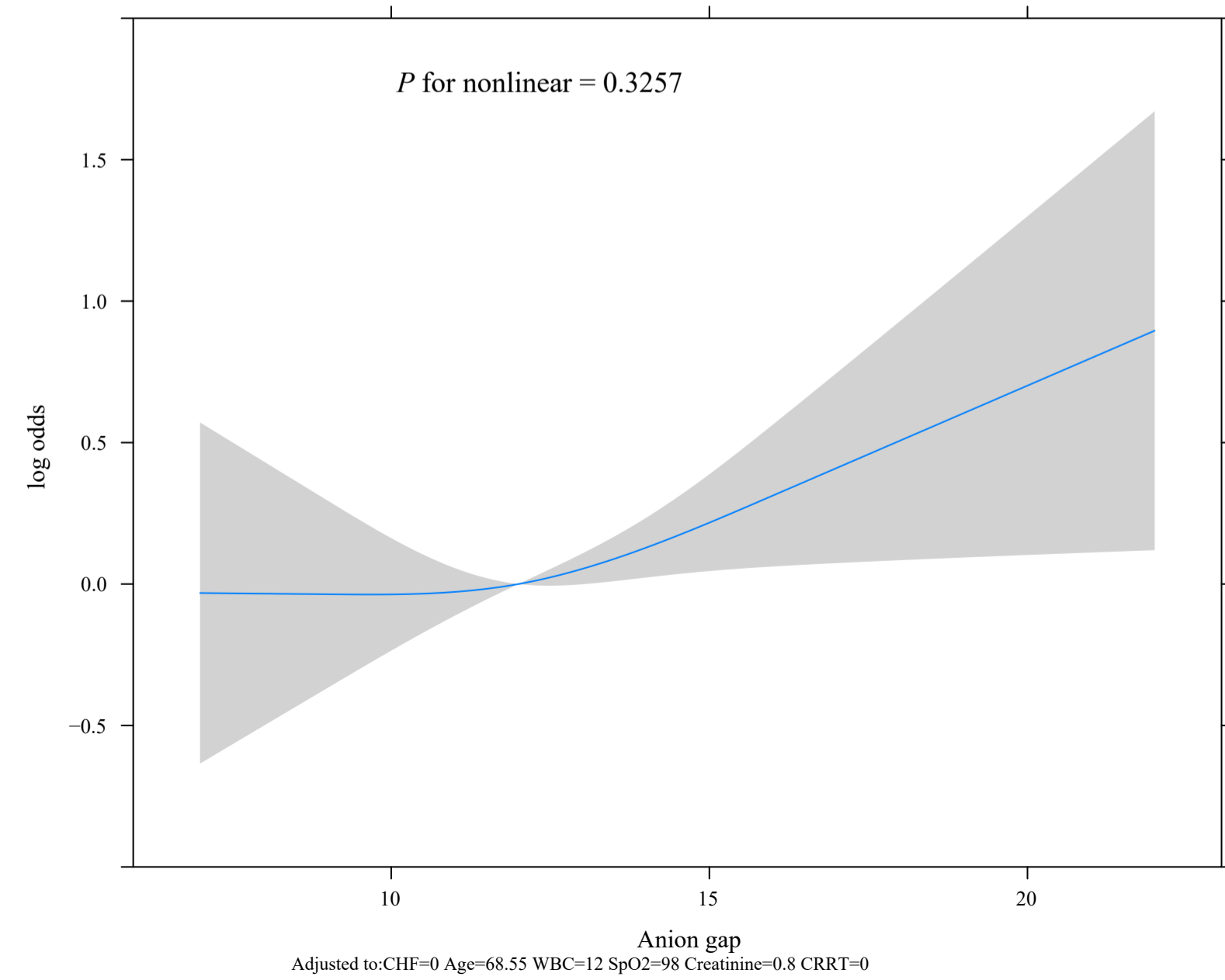

Supplement: Supplementary file 3 — Additional file 3. Loess curves for the correlation between continuous variables and 3-year mortality. [file 12893_2021_1408_MOESM3_ESM.pdf]
