# Supplementary material for: A novel nomogram for predicting 3-year mortality in critically ill patients after coronary artery bypass grafting
Source: BMC Surg. 2021 Nov 30;21:407. doi: 10.1186/s12893-021-01408-8 (PMC8638264; doi:10.1186/s12893-021-01408-8)
Supplement: Supplementary file 2 — Additional file 2. Univariate analyses for the relationship between the candidate risk factors and 3-year mortality in the primary cohort. [file 12893_2021_1408_MOESM2_ESM.docx]

| Additional file 2. Univariate analyses for the relationship between the candidate risk factors and 3-year mortality in the primary cohort | | | |
| --- | --- | --- | --- |
| Characteristics | OR | 95% CI | P |
| Age, years | 1.07 | 1.05-1.08 | <0.001 |
| Gender, female | 1.38 | 1.05-1.83 | 0.022 |
| CHF | 3.74 | 2.87-4.87 | <0.001 |
| Renal failure | 3.29 | 2.27-4.78 | <0.001 |
| Diabetes | 1.05 | 0.81-1.37 | 0.710 |
| Previous myocardial Infarction | 1.09 | 0.79-1.51 | 0.586 |
| Previous stroke | 1.77 | 0.76-4.10 | 0.185 |
| Mean SBP, mmHg | 1.01 | 0.99-1.02 | 0.329 |
| Mean DBP, mmHg | 0.96 | 0.94-0.98 | 0.001 |
| Mean HR, beats/min | 1.00 | 0.99-1.02 | 0.651 |
| Mean Respiratory rate, beats/minute | 1.01 | 0.96-1.05 | 0.764 |
| Mean MBP, mmHg | 1.00 | 0.98-1.02 | 0.982 |
| Hemoglobin, g/dL | 0.92 | 0.85-0.99 | 0.023 |
| WBC, 109/L | 1.03 | 1.00-1.05 | 0.018 |
| APTT, second | 1.02 | 1.01-1.02 | <0.001 |
| Creatinine, U/L | 1.55 | 1.36-1.77 | <0.001 |
| BUN, mg/dL | 1.05 | 1.04-1.06 | <0.001 |
| Platelet, 109/L | 1.00 | 1.00-1.00 | 0.866 |
| PCO2 (mmHg) | 1.00 | 0.98-1.02 | 0.696 |
| PO2 (mmHg) | 1.00 | 1.00-1.00 | 0.291 |
| pH | 0.40 | 0.06-2.86 | 0.363 |
| SpO2 (%) | 0.97 | 0.96-0.98 | <0.001 |
| Anion gap, mmol/L | 1.18 | 1.13-1.24 | <0.001 |
| Potassium, mmol/L | 0.97 | 0.86-1.11 | 0.702 |
| Sodium, mmol/L | 0.96 | 0.92-1.00 | 0.048 |
| Mechanical ventilation | 1.11 | 0.62-2.02 | 0.722 |
| CRRT | 43.75 | 16.77-114.11 | <0.001 |
| Vasopressor use | 1.33 | 0.90-1.96 | 0.147 |
